# Viral Signals in Acute Appendicitis: Direct Tissue Infection, Systemic Triggers, and the Unresolved Biology of Spontaneous Resolution

**DOI:** 10.3390/diseases14100361

**Published:** 2026-10-01

**Authors:** Zelimkhan Berikkhanov, Kseniya Resnina, Zakhar Akulov, Evgeniy Tarabrin, Alexey Shestakov, Vadim Razumovsky, Aleksey Kotelnikov, Andrey Nikolaev, Milena Ivanova, Sara Nourmahal, Sergey Muraviev

**Affiliations:** Department of Hospital Surgery No. 2, Sechenov First Moscow State Medical University ( Sechenov University), 119048 Moscow, Russia; berikkhanov_z_g@staff.sechenov.ru (Z.B.); resnina_k_m@student.sechenov.ru (K.R.); tarabrin_e_a@staff.sechenov.ru (E.T.); shestakov_a_l@staff.sechenov.ru (A.S.); razumovskiy_v_s@staff.sechenov.ru (V.R.); kotelnikov_a_g@staff.sechenov.ru (A.K.); nikolaev_a_m@staff.sechenov.ru (A.N.); ivanova_m_yu_1@staff.sechenov.ru (M.I.); nurmakhal_s@student.sechenov.ru (S.N.); muravev_s_yu@staff.sechenov.ru (S.M.)

**Keywords:** acute appendicitis, viral infection, adenovirus, cytomegalovirus, lymphoid hyperplasia, gut virome, bacteriophages, narrative review

## Abstract

Background/Objectives: Acute appendicitis is readily recognised, but its initiating mechanisms remain uncertain. Viral findings are often interpreted without separating direct appendiceal infection, systemic host responses, clinical phenocopies, and virome effects. We examined the evidence for these scenarios and their possible relationship to spontaneous resolution. Methods: A structured narrative synthesis of 82 publications integrated tissue studies, clinical series, epidemiology, microbiome and virome research, natural history, and treatment outcomes. Results: Direct viral injury is documented in rare clinicopathological settings. Available tissue studies argue against frequent direct adenoviral infection. Systemic infection could provoke lymphoid activation, oedema, or early microvascular injury, but this causal sequence has not been demonstrated in humans. Clinical studies document resolution in selected uncomplicated cases, including recovery without antibiotics, without establishing a viral cause. Conclusions: The framework separates observed findings from competing causal hypotheses. A viral prodrome is a candidate research variable whose independent diagnostic and prognostic value remains unestablished. Prospective studies must link infection chronology to local inflammation and subsequent outcomes before such information can guide treatment. Viral symptoms or positive tests cannot justify withholding indicated antibiotics or surgery.

## 1. Introduction

The surgical manifestations of acute appendicitis are understood far better than its onset. Luminal obstruction provides a persuasive account when an appendicolith or another mechanical barrier is present. Intraluminal pressure then rises, venous and lymphatic drainage deteriorate, the mucosal barrier is damaged, and bacterial invasion intensifies. This sequence is well-supported in some patients, but it does not encompass every form of the disease [1,2,3,4,5,6,7].

The population patterns of uncomplicated and perforated appendicitis can change independently [5,6]. Appendicoliths may also be incidental findings that never lead to inflammation [7]. Bacterial communities vary among patients, stages of disease, and regions within the same appendix [8,9,10,11,12,13,14,15,16,17,18,19,20,21]. A similar clinical picture therefore probably emerges from several trajectories with different initiating events.

**Figure 1:**
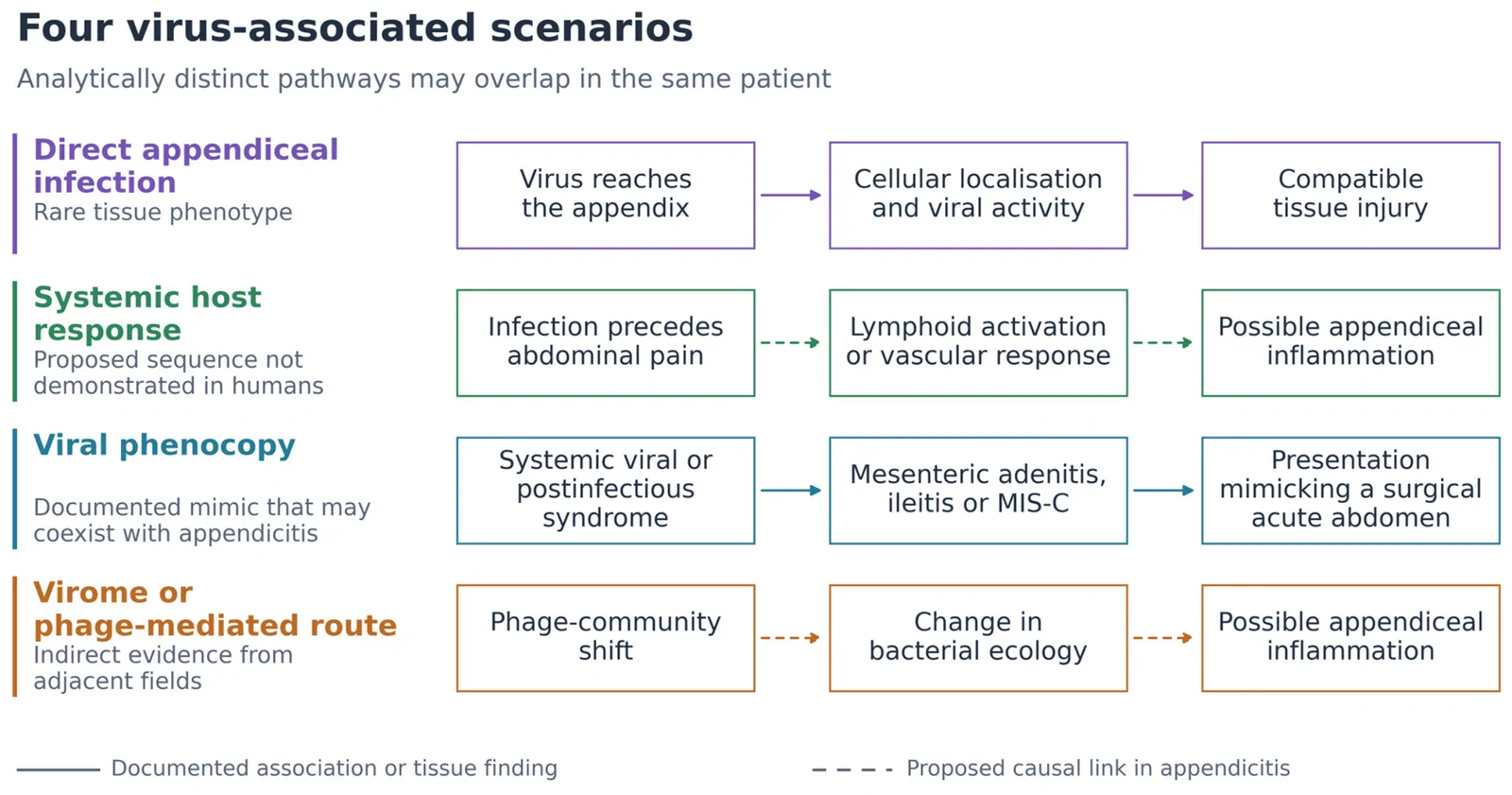
Four virus-associated scenarios in acute appendicitis. Direct infection, systemic triggering, phenocopy, and virome effects are analytically distinct but not mutually exclusive. Solid arrows represent documented associations or tissue findings. Dashed arrows indicate proposed causal links that remain untested in human appendicitis. MIS-C, multisystem inflammatory syndrome in children. Original schematic developed for this review.

The vermiform appendix is rich in lymphoid follicles, forms part of the intestinal immune system, and supports organised biofilms [22,23,24]. During an acute infection, it may respond like other lymphoid tissue. Follicular activation and oedema could transiently narrow the lumen, while systemic inflammation could alter the microcirculation before substantial bacterial invasion develops. If this response subsides before sustained obstruction and destructive injury occur, inflammation might resolve without surgery. This proposed link between systemic infection and reversibility is the central hypothesis of the review. It has not been demonstrated in humans. Figure 1 separates four virus-associated scenarios that require different evidence but may coexist.

A viral signal becomes informative only when its anatomical site and timing are known. A positive tissue polymerase chain reaction (PCR) result may indicate active infection, latency, reactivation, or viral material within the lumen. Evidence of systemic infection does not establish appendiceal involvement. Improvement after analgesia, antibiotics, or observation also cannot identify the initiating mechanism. Similar clinical improvement can accompany transient obstruction, a contained bacterial process, or a sterile immune response.

A systematic review of 19 heterogeneous studies associated appendicitis with adenovirus, herpesviruses, measles, influenza, rotavirus, dengue, and other infections [25]. This list demonstrates the diversity of the observations, not the existence of a single viral form of appendicitis. Some viruses can infect the appendix directly. In a subset of patients, systemic infection may provoke a local immune response. Frequent viral causation of ordinary acute appendicitis has not been demonstrated.

The review examines the evidence for direct viral injury within the appendix, compares systemic immune and microcirculatory hypotheses with obstructive, vascular, and bacterial mechanisms, and asks whether any of these mechanisms can be linked to resolution or progression. Its aim is to identify what is known, which causal steps remain untested, and what evidence would be needed before a viral context could inform clinical decisions.

## 2. Materials and Methods

### 2.1. Design and Source Material

This study was conducted as a structured narrative review. The clinical and biological corpus comprised 82 publications, including tissue studies, retrospective series, epidemiological time-series analyses, case reports, studies of the bacterial microbiome and gut virome, systematic reviews, natural-history studies, and randomised treatment trials. These source types addressed different questions, so no pooled quantitative estimate was calculated. An additional methodological reference informed appraisal of the review during revision.

The original search log, exact search strings, number of retrieved records, and duplicate-removal stages were not retained. No protocol was registered. Study selection and data extraction were not performed by two independent reviewers, and risk of bias was not formally assessed. The manuscript is therefore not presented as a systematic review or a scoping review. The methods are described only to the extent that the procedures can be verified.

During revision, we used the six domains of the Scale for the Assessment of Narrative Review Articles (SANRA) to appraise the review’s importance, aims, search description, referencing, scientific reasoning, and presentation of outcome data [26]. SANRA was used as a critical appraisal tool, not a reporting guideline. The absence of a reproducible search record remained an explicit limitation. No numerical SANRA score was assigned.

### 2.2. Scope

The analysis covered viral genomes, antigens, characteristic morphology, or replication markers detected in the appendix, confirmed systemic infection occurring with appendicitis, viral phenocopies, epidemiological associations, the bacterial microbiome, the gut virome, and the natural history of the disease. Studies of inflammatory bowel disease, general viral ecology, and animal models were used to construct mechanisms. Their findings were not extrapolated to the frequency, prognosis, or treatment of human appendicitis.

The unit of analysis was a discrete signal rather than an entire publication. Signals included cellular viral localisation, a positive PCR result, the co-occurrence of systemic infection and appendicitis, a temporal association, a microbial shift, or a clinical outcome. This approach prevented a small morphological series from becoming an estimate of prevalence and prevented treatment response from being used to infer aetiology.

### 2.3. Causal Interpretation

**Figure 2:**
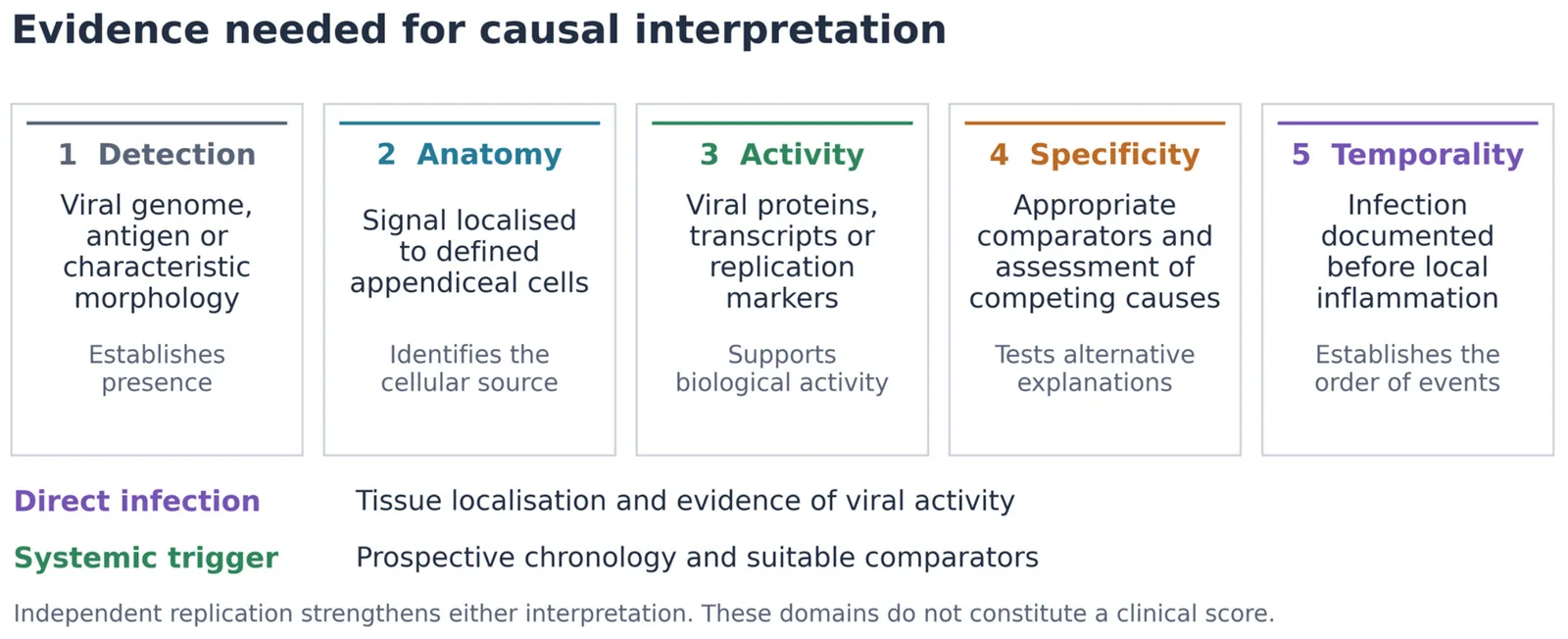
Evidence needed to interpret a viral signal. The five domains organise causal assessment and are not a universal sequence or a clinical score. Direct infection requires cellular localisation in appendiceal tissue and evidence of viral activity. A systemic-trigger hypothesis principally requires prospective chronology and suitable comparator groups. Independent replication strengthens either interpretation. Original schematic developed for this review.

Findings were examined across four scenarios, comprising direct tissue infection, a systemic immune or microcirculatory trigger, a phenocopy, and a possible virome-mediated route. For each finding, we considered the site of detection, evidence of viral activity, temporal order, suitable controls, and competing causes. Primary studies were not assigned an aggregate quality score. Figure 2 sets out the evidence needed to assess these different causal interpretations.

Tissue localisation and activity are central to a claim of direct infection. A systemic-trigger model does not require local replication, but it does require evidence that infection preceded appendiceal inflammation and that the association persists against appropriate comparators. Negative tissue testing alone cannot establish a systemic trigger. These scenarios may overlap in an individual patient. Evidence in the tables is labelled by type, including direct tissue evidence, clinical association, phenocopy evidence, treatment-trajectory evidence, and indirect mechanistic evidence. These categories describe the source and scope of a finding, not a numerical hierarchy of causal certainty. Treatment outcomes were interpreted separately from aetiology.

## 3. Results

### 3.1. Structure of the Evidence Base

The evidence base is markedly uneven. Direct viral injury has been examined in small tissue series and case reports. Systemic infection and phenocopies are described mainly in retrospective studies. Large studies have focused on epidemiology and treatment but have seldom tested appendiceal tissue for viruses. The bacterial microbiome has received far greater attention, whereas almost all evidence on the virome and bacteriophages comes from adjacent fields.

No study in the corpus combined consecutive enrolment, prespecified phenotypes, pretreatment sampling, cellular viral localisation, evidence of replication, suitable controls, and long-term follow-up. Several scenarios remain plausible, but their frequency cannot be estimated from these heterogeneous studies.

### 3.2. The Appendix as an Immune and Microbial Interface

Appendiceal lymphoid follicles are most prominent in children and young adults, the same age groups in which appendicitis is common [1,2,3,22]. This overlap supports an immune hypothesis but does not prove it. Follicular hyperplasia may follow a respiratory or enteric infection, narrow the lumen, accompany local inflammation, or perform more than one of these roles. Distinguishing among them requires a chronology that most studies lack.

The appendiceal mucosa supports biofilms and a microbial community of its own [23,24]. Oedema, impaired drainage, and epithelial injury alter oxygen availability, nutrients, and spatial relationships within this ecosystem. Microbial changes in an inflamed appendix may therefore be a cause, an amplifier, or a consequence of tissue injury. A single postoperative sample cannot establish the order of events.

This anatomy motivates the central working hypothesis. An acute infection outside the appendix could provoke follicular activation, oedema, and transient luminal narrowing without local viral replication. Early microcirculatory injury might contribute in some patients, with bacteria subsequently amplifying inflammation. The sequence remains hypothetical and has not been demonstrated prospectively in humans.

### 3.3. Adenovirus and the Limits of Direct Tissue Evidence

Adenovirus illustrates the divide between appendiceal infection and ordinary suppurative appendicitis. Among 877 paediatric appendicectomies, 94 specimens were clinically suspicious but histologically negative. Lymphoid hyperplasia was present in 63, and 2 showed viral immunoreactivity, epithelial proliferation, and inclusions [27]. Adenovirus can involve the appendix and produce a surgical presentation. This series does not estimate its contribution to typical transmural neutrophilic inflammation.

In 112 appendiceal specimens from young military personnel, quantitative PCR detected adenoviral deoxyribonucleic acid (DNA) in 16 cases, or 14%. Immunohistochemistry was positive in only 1 specimen [28]. The sensitivity of PCR permits the detection of small amounts of genome but does not identify the cellular source. This population also had unusually high adenoviral exposure. In a retrospective series of 277 paediatric appendicectomies, adenovirus was detected in 5.4% of cases and rotavirus in 4.7%, while bacteria were more frequent [29]. Neither study had comparable non-inflamed tissue, and testing conditions differed.

In a consecutive paediatric series, immunohistochemistry was negative for adenovirus in all 421 specimens with acute appendicitis. Among 19 appendices removed during surgery for ileocolic intussusception without appendicitis, 7 were positive and showed epithelial cytopathic changes [30]. This negative result argues against frequent immunohistochemically detectable adenoviral infection in that appendicitis cohort. The intussusception group, however, may have been enriched for adenovirus-associated lymphoid hyperplasia and is not an unbiased estimate of background infection. Positive staining in this selected group demonstrates detection in affected tissue, but does not establish diagnostic sensitivity, specificity, or population prevalence. Negative staining cannot exclude an earlier cleared infection or a systemic response without local antigen.

### 3.4. Cytomegalovirus and Other Herpesviruses

The strongest cytomegalovirus signal came from a study of 14 immunocompetent patients. Early antigen together with interleukin-6 or interleukin-8 was found in 9 specimens, and late antigen in 6. Active antigens were absent from 6 non-inflamed appendices, although some controls had viral DNA or serological evidence of prior infection [31]. Cellular localisation, markers of activity, and tissue controls strengthen this result. Its small sample and high proportion of positive findings call for independent replication.

In a series of 38 children with advanced or perforated inflammation, cytomegalovirus was detected most often, followed by human herpesvirus 6. Viral DNA was found in the appendix, omentum, and blood [32]. A signal across several anatomical sites is compatible with systemic infection or reactivation during severe inflammation. Healthy children’s blood served as the control, but non-inflamed appendices were not studied. Local causation cannot be separated from systemic accompaniment in this design.

Case reports document rare cytomegalovirus appendicitis in immunocompetent patients, after acute Epstein–Barr virus infection, and in solid-organ transplant recipients [33,34,35,36]. Infectious mononucleosis has also been associated with histologically confirmed inflammation and Epstein–Barr virus-encoded small ribonucleic acid (EBER)-positive tissue [37,38]. Small series and case reports of varicella-zoster virus describe viral DNA or characteristic inclusions in the appendix [39,40,41]. These reports establish biological possibility, but their selection for unusual presentations precludes any estimate of frequency.

### 3.5. Measles, Influenza, and Rotavirus

Measles-associated appendicitis is rare but morphologically distinctive. Stage-dependent tissue changes, involvement of immune cells, and a viral signal have been described [42,43]. The concurrence of systemic measles with a characteristic tissue pattern supports direct involvement in isolated cases. This narrow phenotype cannot be generalised to routine appendicitis.

Cases of appendicitis during confirmed or probable influenza A(H1N1) infection have been reported in adults and children [44,45,46]. The virus was generally not localised in appendiceal tissue. These observations are compatible with a systemic immune trigger, but they also permit a chance concurrence of two common events. The proposed association with rotavirus likewise rests on isolated findings and retrospective detection, not on consecutive tissue studies.

A population analysis of United States hospital admissions from 1970 to 2006 found none of the seasonal pattern expected if influenza or rotavirus commonly infected the appendix directly [47]. Rotavirus was not associated with non-perforated appendicitis. This finding substantially weakens a model centred on one prevalent pathogen. It does not test whether different viruses in different seasons elicit a similar systemic response in a small subgroup. That hypothesis requires patient-level chronology and laboratory confirmation rather than the coincidence of seasonal curves.

### 3.6. Severe Acute Respiratory Syndrome Coronavirus 2 (SARS-CoV-2) and Multisystem Inflammatory Syndrome

The coronavirus disease 2019 (COVID-19) pandemic provided a natural test of the infectious hypothesis while introducing substantial confounding. Access to care, time to presentation, thresholds for imaging, antibiotic use, and operative practice all changed appreciably [48,49,50,51]. A higher proportion of complicated cases during this period cannot automatically be attributed to SARS-CoV-2 biology.

Acute appendicitis has been reported in adults with COVID-19. In one paediatric case, reverse-transcription quantitative PCR detected SARS-CoV-2 ribonucleic acid (RNA) in the resected appendix [48,49]. The child also had an appendicolith, perforation, and transmural neutrophilic inflammation. The tissue result confirms viral presence, but mechanical obstruction and bacterial complications remain more immediate explanations. Cellular localisation and active replication were not assessed.

Multisystem inflammatory syndrome in children after SARS-CoV-2 is an important clinical phenocopy. Right-sided pain, bowel-wall thickening, mesenteric lymphadenitis, and marked systemic inflammation may lead to surgery. A systematic review found that the syndrome often mimics a surgical acute abdomen and sometimes coexists with true appendicitis [51]. Assessment must therefore consider both the inflammatory syndrome and a surgical cause. These findings are specific to a defined paediatric syndrome and cannot be extrapolated directly to ordinary appendicitis.

### 3.7. Systemic Infection and a Local Response as a Working Hypothesis

The proposed systemic sequence begins with an acute respiratory or enteric infection. Subsequent lymphoid activation and oedema could transiently narrow the appendiceal lumen, impair drainage, and initiate local inflammation without viral replication in the appendix. Appendiceal immune anatomy and reports of lymphoid hyperplasia provide indirect support for this possibility [22,27,28,29,31,32]. Tissue infection and systemic triggering nevertheless remain different explanations. These observations do not demonstrate that infection elsewhere causes appendiceal inflammation or a resolving clinical course.

A second branch of the hypothesis involves the microcirculation. Altered endothelial Fas-ligand signalling and changes in the fibrinolytic mediators urokinase-type plasminogen activator (uPA) and plasminogen activator inhibitor 1 (PAI-1) have been described in inflamed tissue [52,53]. These findings confirm vascular involvement after the process is established but do not establish temporal order. Influenza reports document the concurrence of infection and appendicitis, yet they do not demonstrate virus within the endothelium or appendiceal tissue [44,45,46]. A virus-induced appendiceal endotheliopathy remains an extrapolation.

Testing this route requires documentation of infection before abdominal pain, early assessment of local inflammation, and adjustment for appendicoliths and other competing causes. Comparator groups should include appendicitis without a preceding viral illness, viral illness without abdominal pain, phenocopies, and non-inflamed appendices where tissue is available. Prospective chronology is needed to distinguish a preceding systemic response from inflammation caused by established appendicitis.

### 3.8. Evidence from Human Studies

Table 1 summarises key human studies by evidence type, observed finding, and limit of causal inference. Tissue detection is distinguished from cellular localisation and activity, and from clinical or epidemiological association.

### 3.9. Bacteria as a Competing Mechanism and a Possible Second Hit

Bacterial invasion remains the best-developed microbial explanation. In situ hybridisation showed deep penetration of *Fusobacterium nucleatum* and *Fusobacterium necrophorum* into inflamed tissue, with no comparable signal in controls [9,10]. Studies using 16S ribosomal RNA (rRNA) sequencing reported enrichment of *Fusobacterium*, *Porphyromonas*, *Parvimonas*, *Gemella*, and other oral taxa [11,12,13]. Several studies found different bacterial profiles in uncomplicated and complicated disease [14,15]. Spatial invasion provides stronger causal support than routine culture.

No single bacterial signature has emerged. In a prospective paediatric series of 22 patients, differences between groups remained trends against substantial within-group variability. *Fusobacterium* increased in phlegmonous and perforated phenotypes but not in gangrenous disease. The result varied with sampling site and obstruction [16]. Other studies reported shifts in innate immunity, *Campylobacter jejuni*, dysbiosis, and links to the oral microbiota, but did not reproduce one profile [17,18,19,20,21]. A systematic review of culture-independent studies likewise found no reliable discriminator between uncomplicated and complicated appendicitis [8].

Bacteria may initiate disease or amplify an earlier process. A systemic immune response or transient obstruction could alter the mucosal barrier, allowing bacterial invasion to drive suppurative progression. Because appendices are usually sampled after inflammation is established and often after antibiotics, surgical specimens rarely resolve the order of these events.

### 3.10. The Virome and Bacteriophages as an Untested Mechanism

The gut virome contains eukaryotic viruses, bacteriophages, and many unannotated sequences. In healthy people, it is individual-specific and relatively stable, yet it responds to age, diet, medication, and inflammation [54,55,56,57,58,59,60]. Inflammatory bowel disease has been associated with shifts in phage communities and their bacterial relationships. Experimental colitis also produces a disease-associated phage configuration [61]. None of these studies prospectively examined the appendiceal virome during appendicitis.

A phage-mediated change in biofilm organisation is one possible explanation for shifts in the appendiceal bacterial community. Such a change could precede bacterial invasion, but it could also follow inflammation, bacterial lysis, or antibiotic exposure. No specific, reproducible phage pattern has been established in appendicitis.

This mechanism merits separate study because it connects two observed facts. The appendix supports biofilms, and bacterial communities in inflamed tissue vary across patients and phenotypes. A causal test must show that a phage change precedes a shift in defined bacteria and accompanies a specific tissue injury. Until then, the virome generates hypotheses but provides no basis for antiviral or phage-based treatment.

### 3.11. Obstruction and Vascular Injury as Essential Competing Mechanisms

An appendicolith is an important marker of a possible obstructive mechanism. It does not invariably cause inflammation, but in established appendicitis it is associated with greater severity and poorer outcomes after non-operative treatment [7,62,63,64]. Analyses of viral signals must therefore account for appendicoliths as a competing explanation and a modifier of disease course.

Vascular injury, endothelial activation, and impaired fibrinolysis are documented in acute appendicitis [52,53]. They are compatible with an ischaemic component, particularly in severe inflammation and patients with vascular risk. The evidence is insufficient to regard primary infarction as a common independent cause. Necrosis may arise from pressure, venous congestion, inflammation, thrombosis, or a combination of these processes. A primary vascular phenotype would require early perfusion assessment, standardised vascular histology, and clinical data obtained before necrosis develops.

Table 2 compares the evidence supporting each mechanism with observations that could distinguish it from competing explanations. The predictions are proposed tests, not findings already established by the review.

### 3.12. Spontaneous Resolution Exists, but Its Cause Is Unknown

Randomised trials provide reliable evidence on treatment but reveal little about the initiating mechanism. The Comparison of Outcomes of Antibiotic Drugs and Appendectomy (CODA) trial enrolled 1552 adults. Antibiotics were non-inferior to surgery for 30-day general health status, although 29% of patients assigned to antibiotics underwent appendicectomy within 90 days. Outcomes were poorer in the presence of an appendicolith [63]. The Appendicitis Acuta (APPAC) trial showed that immediate surgery can be avoided in selected adults with computed tomography (CT)-confirmed uncomplicated disease without an appendicolith [65]. Reports at 5 and 10 years followed the original randomised cohort [66,67]. At 10 years, histologically confirmed recurrence was 37.8% and the cumulative appendicectomy rate was 44.3% [67]. These outcomes describe recovery after antibiotic treatment and cannot, by themselves, establish spontaneous resolution.

An individual-patient-data meta-analysis of 6 randomised trials included 2101 adults. Within 1 year, 33.9% of patients initially treated with antibiotics had undergone surgery. The rate reached 48.7% among patients with an appendicolith, who also had more complications [64]. Other meta-analyses report a similar pattern [68,69]. A substantial proportion of carefully selected adults avoid surgery during the first year, but the risks of failure and later appendicectomy remain material.

APPAC III addressed spontaneous resolution more directly. This separate, double-blind superiority trial assigned adults with mild, CT-confirmed uncomplicated appendicitis to antibiotics or placebo. Of 72 randomised patients, 66 were analysed. By day 10, treatment had succeeded in 87% of the placebo group and 97% of the antibiotic group. The small trial did not demonstrate antibiotic superiority and was not designed to establish equivalence [70]. Three-year follow-up of the same cohort likewise did not establish an antibiotic advantage [71]. Recovery without antibiotics occurred in selected patients, but its aetiology was not determined.

Paediatric treatment outcomes require separate assessment. In an international randomised trial of 936 children aged 5–16 years, 1-year treatment failure occurred in 34% after antibiotics and 7% after appendicectomy. The antibiotic strategy did not meet the prespecified criterion for non-inferiority [72]. Other comparative studies show that some children avoid immediate surgery, while recurrence, failure, and appendicoliths remain important [73,74,75,76,77]. Age alone does not identify a viral or benign form of appendicitis. Table 3 distinguishes these treatment trajectories from evidence about aetiology and identifies reports from the same trial cohort.

### 3.13. Infectious History as a Testable Clinical Feature

Symptom chronology can restore information that postoperative tissue has already lost. Fever, chills, myalgia, sore or scratchy throat, rhinorrhoea, cough, and diarrhoea should be recorded separately when they precede pain in the right iliac fossa. Such features direct attention towards respiratory or enteric infection, mesenteric lymphadenitis, terminal ileitis, and multisystem inflammatory syndrome in children [44,45,46,47,48,49,50,51]. In an immunocompromised patient, they also increase concern for tissue-invasive cytomegalovirus [33,34,35,36].

Such a history may broaden the differential diagnosis and support prospective phenotyping, but its independent diagnostic and prognostic value has not been established. A prodrome is common, often recalled retrospectively, and may be unrelated to appendiceal inflammation. Its absence also cannot exclude a preceding infection.

Treatment response is even less specific. Analgesics and anti-inflammatory drugs reduce pain arising from different mechanisms. Antibiotics may suppress secondary bacterial invasion even when an immune event came first. Observation allows transient obstruction or limited inflammation time to resolve. Local cooling has no established diagnostic or therapeutic value in appendicitis. Improvement over several hours does not distinguish viral, bacterial, obstructive, and sterile immune processes.

Clinical guidelines base non-operative management on assessment of disease severity, imaging when indicated, repeat examination, and prompt escalation if the patient deteriorates [78,79]. Appendicoliths and other predictors of failure require specific consideration. A preceding viral illness is not part of validated selection criteria.

Future studies can record prodromal features alongside imaging and serial clinical change. These features do not justify treating presumed viral appendicitis with non-steroidal anti-inflammatory drugs and cooling alone, or withholding indicated antibiotics or surgery.

## 4. Discussion

### 4.1. Principal Finding

The evidence supports rare direct viral injury and clinically important phenocopies. It does not establish a common viral cause of ordinary acute appendicitis. The main contribution of this review is to separate these findings from the proposed systemic and virome-mediated mechanisms, which require different tests. The link between a systemic infection and reversible appendiceal inflammation remains an unconfirmed hypothesis.

Appendiceal immune anatomy makes a systemic mechanism plausible. Lymphoid activation could narrow the lumen, and an early vascular response could impair perfusion before extensive bacterial invasion [22,23,24,52,53]. In this proposed model, systemic viral infection could initiate appendiceal inflammation without local replication. This causal sequence has not been demonstrated in humans, nor has it been linked prospectively to spontaneous resolution.

### 4.2. Interpreting Conflicting Evidence

The adenovirus studies answer different questions. Selected specimens with lymphoid hyperplasia demonstrate epithelial viral injury, whereas PCR-positive appendices establish the presence of viral DNA without necessarily showing its cellular source or activity [27,28,29]. Negative immunohistochemistry in 421 consecutive cases argues against frequent detectable adenoviral infection in typical paediatric appendicitis [30]. Its intussusception controls may have been enriched for adenoviral disease, so they cannot define background prevalence or assay sensitivity. These findings constrain a common direct-infection model but neither confirm nor exclude a systemic trigger.

Cytomegalovirus, other herpesviruses, measles, and varicella define a different evidential boundary. Cellular localisation, markers of activity, and concordant morphology can establish a direct phenotype in one patient or a small series. Selective publication of unusual cases cannot establish how frequent that phenotype is [31,32,33,34,35,36,37,38,39,40,41,42,43]. The direct mechanism exists, but occupies a narrow clinical niche.

The absence of a seasonal association with influenza and rotavirus also needs a precise interpretation [47]. It weakens the proposition that one common virus frequently infects the appendix. A population time series cannot determine whether different infections at different times elicit a similar host response. That question requires an individual chronology recorded before treatment.

### 4.3. The Viral Model Among Competing Mechanisms

A proposed systemic trigger must be evaluated against stronger competing explanations. Bacterial invasion is spatially documented, appendicoliths predict poorer non-operative outcomes, and vascular injury accompanies established inflammation [7,8,9,10,11,12,13,14,15,16,17,18,19,20,21,52,53,62,63,64]. The presence of a viral illness cannot displace these mechanisms. Its causal relevance would require an association that precedes local injury and remains after adjustment for them.

These mechanisms may act alone or in sequence. A resected appendix reveals the endpoint, not the timing of the first injury. The systemic model would become persuasive if confirmed infection preceded pain, an early lymphoid or vascular phenotype followed, and the association survived adjustment for appendicoliths, bacterial invasion, age, and treatment. Loss of the association after such adjustment would argue directly against the model.

The virome hypothesis is less directly supported. Associations in inflammatory bowel disease and experimental colitis suggest that phages can influence bacterial ecology, but they do not establish such a sequence in appendicitis [54,55,56,57,58,59,60,61]. Longitudinal sampling would need to show whether a phage change precedes bacterial invasion or follows inflammation and treatment.

### 4.4. Spontaneous Resolution and Managed Risk

Non-operative studies demonstrate that selected episodes resolve without immediate surgery, while APPAC III also documents recovery without antibiotics. These observations establish variation in clinical course, not a viral aetiology [63,64,65,66,67,68,69,70,71,72,73,74,75,76,77]. Antibiotic-treated recovery must be distinguished from spontaneous resolution. Neither outcome identifies the initiating immune, obstructive, vascular, or bacterial mechanism.

A viral prodrome, imaging without an appendicolith or complication, and early improvement are candidate research variables. They are not validated markers of a reversible immune phenotype. A positive viral test outside the appendix likewise cannot distinguish resolution from dangerous progression. A virus-associated phenotype therefore remains a subject for prospective study, not a clinical diagnosis.

Longitudinal assessment could test whether infectious history adds information beyond imaging and serial examination. The present evidence does not establish a virus-specific monitoring schedule, diagnostic threshold, or stopping rule. Any prospective study would need to prespecify these procedures within an established clinical care pathway and validate their performance separately.

Current decisions about imaging, antibiotics, observation, and surgery should continue to follow established assessment and treatment standards [78,79,80,81,82,83]. A viral prodrome alone does not reduce the risk of progression or negate an indication for treatment. Less invasive care could be investigated only if a reproducible subgroup is first shown to have a sufficiently low risk under clearly defined conditions.

### 4.5. Limitations

The original search log was not retained, so the search cannot be reproduced and the completeness of the corpus cannot be measured. Selection and data extraction were conducted without independent duplicate review, and primary-study risk of bias was not formally assessed. Appraisal against the SANRA domains improved transparency but cannot remedy these limitations. Selection may have favoured unusual positive cases and the authors’ interpretation.

The primary studies differed in age, immune status, virus, specimen, assay, and disease stage. Tissue was usually obtained after a surgical presentation had developed and often after antibiotics. Latency and reactivation complicate interpretation of herpesvirus PCR. Many studies lacked non-inflamed tissue, systemic comparator groups, cellular localisation, or evidence of replication. The four-part framework is an author-derived analytical model. It cannot estimate prevalence or classify an individual patient.

The proposed connection between systemic viral infection and a reversible immune phenotype is speculative and represents the authors’ hypothesis. No prospective study in the corpus demonstrates that systemic infection initiates appendiceal inflammation or predicts its resolution. Rare direct tissue infections do not validate this systemic sequence, and recovery without surgery does not identify a viral cause. The absence of tissue from most non-operative cases further limits comparison with surgical phenotypes.

### 4.6. From Hypothesis to Test

**Figure 3:**
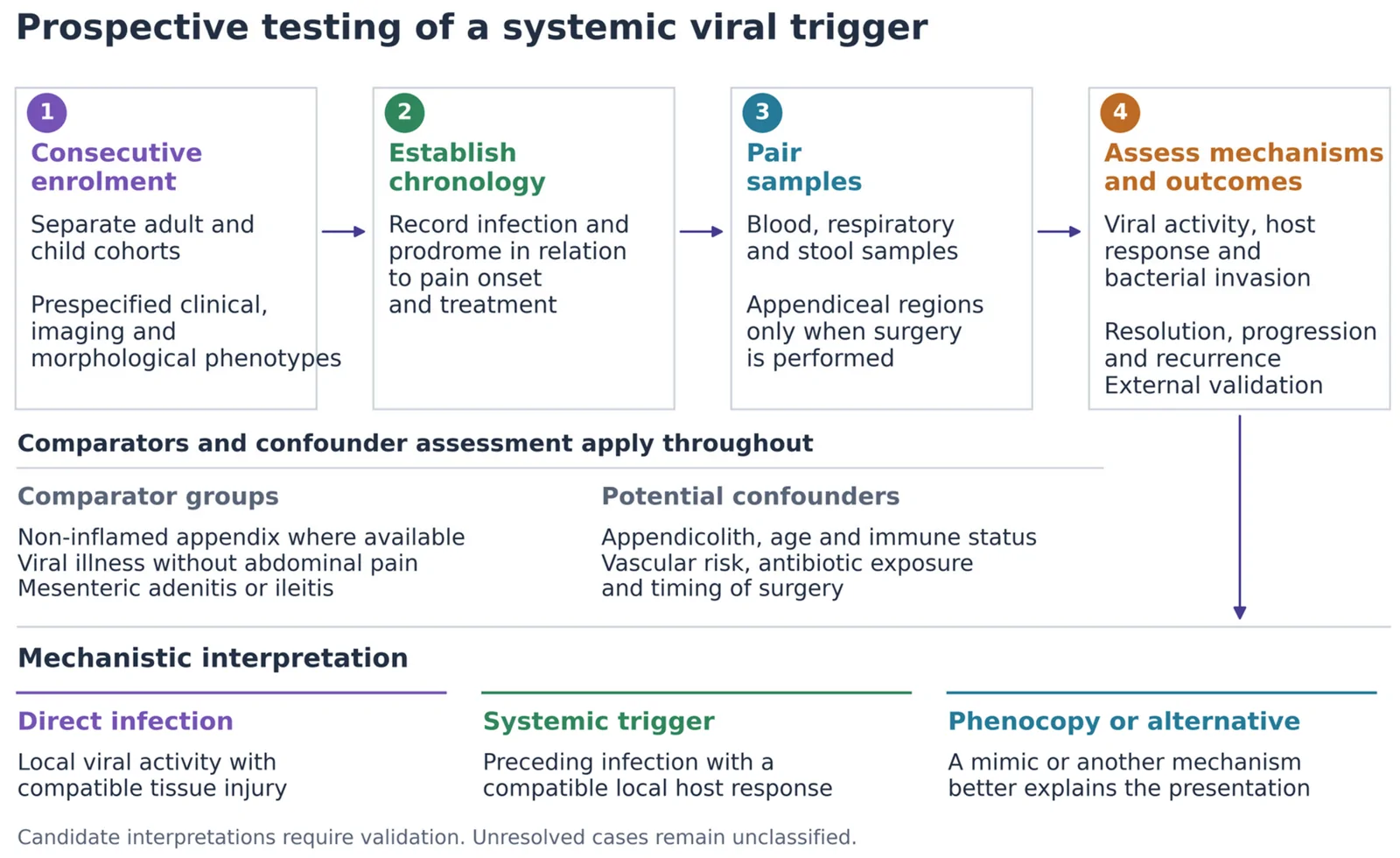
Proposed four-stage study of a systemic viral trigger. Comparators and confounder assessment apply throughout. The authors’ hypothesis remains unconfirmed, and this framework is not a diagnostic or treatment protocol. Appendiceal tissue is usually available only after surgery, limiting direct aetiological comparison with patients who recover without surgery. Original schematic developed for this review.

A prospective study could test the systemic hypothesis by establishing temporal order and comparing it with alternative mechanisms. Figure 3 sets out four sequential stages, with comparator groups and confounder assessment throughout.

A study should address the following priorities.

- Recruit consecutive multicentre cohorts, analyse adults and children separately, and prespecify clinical, imaging, and morphological phenotypes.
- Record the infectious prodrome in relation to pain onset and treatment, with paired blood, respiratory, and stool sampling where feasible without delaying care.
- When surgery is performed, sample appendiceal regions separately for quantitative PCR, in situ localisation, markers of viral activity, and standardised histology.
- Include appropriate comparator groups and account for bacterial invasion, the virome, appendicoliths, perfusion, age, immune status, vascular risk, and treatment timing.
- Prespecify resolution, necrosis, perforation, and recurrence as outcomes, and seek external validation before using a proposed phenotype to guide treatment.

The decisive test is whether a preceding infection explains local inflammation or improves prediction after competing causes have been considered. A reproducible association would justify further work on mechanism and patient selection. A negative result would narrow the hypothesis and prevent a plausible narrative from influencing care without evidence.

## 5. Conclusions

Rare direct viral injury and clinically important phenocopies are documented, but viruses have not been established as a common cause of ordinary acute appendicitis. A systemic infection could, in principle, provoke lymphoid or early microcirculatory changes without local replication. This causal sequence and its proposed link to spontaneous resolution remain unconfirmed in humans.

A structured infectious history may broaden the differential diagnosis and support prospective phenotyping, but its independent diagnostic and prognostic value has not been established. Viral symptoms or positive tests cannot replace imaging, serial examination, or indicated antibiotic and surgical treatment.

Prospective studies can now address a focused question. Does a documented infection precede a reproducible pattern of appendiceal inflammation and predict its course beyond established clinical factors? Answering it would provide a sound basis for more precise patient selection and, if supported by subsequent trials, less invasive care.

## Figures and Tables

**Table 1:** Human studies of viral signals and their limits of causal inference.

Viral Signal	Evidence Type	Design and Method	Observed Finding	Limit of Inference
Adenovirus	Direct tissue evidence	63 paediatric appendices with lymphoid hyperplasia. Histology and immunohistochemistry [27]	Viral immunoreactivity, epithelial proliferation, and inclusions in 2 specimens	Rare tissue phenotype. Frequency in typical suppurative appendicitis cannot be estimated
Adenovirus	Direct tissue evidence, predominantly genome detection	112 appendicectomies in young military personnel. Quantitative PCR and immunohistochemistry [28]	PCR positive in 16 specimens. Immunohistochemistry positive in 1	PCR alone does not localise the signal or establish activity. Highly exposed population
Mixed pathogens	Clinical association	277 paediatric appendicectomies. Retrospective virological and bacteriological testing [29]	Adenovirus detected in 5.4% and rotavirus in 4.7%. Bacteria were more frequent	Co-detection does not establish the initiating event or a common mechanism
Adenovirus	Direct tissue evidence, negative in the appendicitis cohort	421 consecutive paediatric appendicitis specimens and 19 intussusception controls. Immunohistochemistry [30]	No positive appendicitis specimens. Signal and cytopathic change in 7 controls	Constrains detectable direct infection. Controls may be enriched for adenoviral disease and do not estimate background prevalence
Cytomegalovirus	Direct tissue evidence with activity markers	14 immunocompetent patients and 6 non-inflamed controls. Early and late antigens [31]	Early antigen in 9 and late antigen in 6 appendicitis specimens. Active antigens absent in controls	Small series requiring independent replication. Does not establish population prevalence
Herpesviruses	Direct tissue evidence of genome detection and clinical association	38 children with advanced or perforated inflammation. PCR of appendix, omentum, and blood [32]	Cytomegalovirus and HHV-6 detected across anatomical sites	Local infection cannot be separated from systemic infection or reactivation. No non-inflamed appendix controls
Influenza and rotavirus	Clinical association at population level	US hospital-admission time series, 1970–2006 [47]	No seasonal concordance expected for a common direct cause	Ecological data do not establish individual chronology or test a systemic trigger
SARS-CoV-2	Clinical association and direct tissue evidence of RNA detection	Adult clinical reports and a paediatric tissue-PCR case [48,49]	Appendicitis during COVID-19. Viral RNA detected in one resected appendix	No cellular localisation or replication evidence. Appendicolith and perforation confounded the tissue-positive case
MIS-C	Phenocopy evidence	Systematic review of acute abdominal presentations [51]	MIS-C can mimic a surgical acute abdomen and coexist with appendicitis	A syndrome-specific phenocopy cannot establish viral causation of ordinary appendicitis

Evidence types describe what was measured, not proof of causation. Direct tissue evidence includes negative assays and genome detection without demonstrated activity. HHV-6, human herpesvirus 6. MIS-C, multisystem inflammatory syndrome in children. PCR, polymerase chain reaction. COVID-19, coronavirus disease 2019. RNA, ribonucleic acid. SARS-CoV-2, severe acute respiratory syndrome coronavirus 2. US, United States. Original synthesis of the cited studies.

**Table 2:** Evidence supporting competing mechanisms and predictions for future testing.

Mechanism	Current Evidence Type	Predicted Pattern	Discriminating Observation	Finding That Would Weaken It
Direct viral infection	Direct tissue evidence in rare settings	Viral activity in appendiceal cells precedes substantial neutrophilic injury	Cellular localisation, activity or replication markers, and suitable tissue controls	Equal background signal in controls, no activity, or signal confined to the lumen
Systemic immune trigger	Clinical association and indirect mechanistic evidence	Confirmed infection precedes pain, followed by follicular activation and oedema	Prospective chronology, paired systemic samples, and early lymphoid histology	Infection begins after pain or the association disappears after adjustment for competing causes
Systemic vascular trigger	Indirect mechanistic evidence	Infection precedes perfusion impairment or endothelial activation	Early perfusion assessment, vascular histology, and investigational endothelial markers	Vascular changes follow pressure, necrosis, or established inflammation
Primary bacterial invasion	Direct tissue evidence and clinical association	Bacterial penetration precedes deep wall injury	Pretreatment bacterial load and localisation compared with appropriate controls	Early injury occurs without invasion or the bacterial shift appears only later
Obstruction	Clinical association and treatment-trajectory evidence	An appendicolith or another barrier impairs drainage and shapes tissue injury	Concordant imaging, gross anatomy, and histology at the site of obstruction	No obstruction or a barrier without corresponding injury
Primary vascular injury	Direct tissue evidence of injury with indirect evidence of initiation	Perfusion impairment or thrombosis precedes inflammatory necrosis	Perfusion imaging and early vascular histology, with adjustment for vascular risk	Ischaemia appears late and is explained by pressure or inflammation
Viral phenocopy	Phenocopy evidence	A surgical presentation occurs without typical transmural neutrophilic inflammation	Histology where available and comparators with mesenteric adenitis, ileitis, or MIS-C	Confirmed appendicitis better explains the presentation, although coexistence remains possible
Phage-mediated dysbiosis	Indirect mechanistic evidence from adjacent fields	A phage-community shift precedes a change in defined bacteria	Longitudinal virome and bacterial profiling with load assessment	Both communities change only after inflammation or antibiotic exposure

Evidence types refer to the existing literature discussed in Section 3.3, Section 3.4, Section 3.5, Section 3.6, Section 3.7, Section 3.8, Section 3.9, Section 3.10 and Section 3.11. Predicted patterns and discriminating observations are proposals for testing, not demonstrated sequences. The mechanisms may overlap. MIS-C, multisystem inflammatory syndrome in children. Original analytical synthesis.

**Table 3:** Treatment trajectories and the limits of aetiological inference.

Study and Cohort	Evidence Type	Clinical Observation	Supported Inference	Limit of Inference
CODA [63]	Treatment-trajectory evidence	29% of antibiotic-assigned adults underwent appendicectomy within 90 days	Some patients avoid early surgery, with greater failure risk when an appendicolith is present	Response to antibiotics does not identify the initiating aetiology
Individual-patient-data meta-analysis of 6 trials [64]	Treatment-trajectory evidence	Appendicectomy within 1 year in 33.9% overall and 48.7% with an appendicolith	Appendicolith status modifies outcome after initial antibiotic treatment	Pooled treatment outcomes cannot establish a single mechanism and may include cohorts reported separately
APPAC primary trial [65] with 5-year [66] and 10-year [67] follow-up	Treatment-trajectory evidence from one original randomised cohort	At 10 years, confirmed recurrence was 37.8% and cumulative appendicectomy 44.3%	Durable recovery after antibiotics is possible in selected adults without an appendicolith	Follow-up reports are not independent trials. Non-operative recovery does not establish spontaneous or viral resolution
APPAC III primary trial [70] and 3-year follow-up [71]	Treatment-trajectory evidence from a separate trial and its follow-up	Day-10 success was 87% with placebo and 97% with antibiotics. Follow-up involved the same cohort	Resolution without antibiotics occurred in selected adults with mild CT-confirmed disease	The small superiority trial did not establish equivalence. Resolution does not prove a viral cause
International paediatric randomised trial [72]	Treatment-trajectory evidence	At 1 year, treatment failure was 34% after antibiotics and 7% after appendicectomy	The antibiotic strategy failed to meet the prespecified non-inferiority criterion	Trial-defined treatment failure does not identify aetiology. Childhood is not a marker of a benign viral form

CODA, Comparison of Outcomes of Antibiotic Drugs and Appendectomy. APPAC, Appendicitis Acuta. APPAC and APPAC III are separate trials. Each trial’s primary report and follow-up publications describe the same original cohort and must not be counted as independent randomised studies. Outcomes and treatment-failure definitions differ across studies. CT, computed tomography. Original synthesis of the cited studies.

## Data Availability

No new data were created or analysed in this study. Data sharing is not applicable to this article.
